# Chemical Profile and Healthy Properties of Sicilian *Diplotaxis harra* subsp. *crassifolia* (Raf.) Maire

**DOI:** 10.3390/molecules29112450

**Published:** 2024-05-23

**Authors:** Natale Badalamenti, Assunta Napolitano, Maurizio Bruno, Roberta Pino, Rosa Tundis, Vincenzo Ilardi, Monica Rosa Loizzo, Sonia Piacente

**Affiliations:** 1 Department of Biological, Chemical and Pharmaceutical Sciences and Technologies (STEBICEF), University of Palermo, Viale delle Scienze, 90128 Palermo, PA, Italy; natale.badalamenti@unipa.it (N.B.); vincenzo.ilardi@unipa.it (V.I.); 2 NBFC-National Biodiversity Future Center, Piazza Marina 60, 90133 Palermo, PA, Italy; piacente@unisa.it; 3 Department of Pharmacy, University of Salerno, 84084 Fisciano, SA, Italy; anapoli@unisa.it; 4 Centro Interdipartimentale di Ricerca “Riutilizzo Bio-Based Degli Scarti da Matrici Agroalimentari” (RIVIVE), University of Palermo, Viale delle Scienze, 90128 Palermo, PA, Italy; 5 Department of Pharmacy, Health and Nutritional Sciences, University of Calabria, 87036 Arcavacata di Rende, CS, Italy; robertapino95@gmail.com (R.P.); rosa.tundis@unical.it (R.T.); monica_rosa.loizzo@unical.it (M.R.L.)

**Keywords:** antioxidant activity, carbohydrate-hydrolyzing enzymes, glucosinolate, lipase inhibition, wild-wall rocket salad

## Abstract

This study was aimed at investigating the phytochemical profile and bioactivity of *Diplotaxis harra* subsp. *crassifolia* (Brassicaceae), a species from central–southern Sicily (Italy), where it is consumed as a salad. For this purpose, LC–ESI/HRMS^n^ analysis of the ethanolic extract was performed, highlighting the occurrence, along with flavonoids, hydroxycinnamic acid derivatives, and oxylipins, of sulfated secondary metabolites, including glucosinolates and various sulfooxy derivatives (e.g., C13 nor-isoprenoids, hydroxyphenyl, and hydroxybenzoic acid derivatives), most of which were never reported before in the Brassicaeae family or in the *Diplotaxis* genus. Following ethnomedicinal information regarding this species used for the treatment of various pathologies such as diabetes and hypercholesterolemia, *D. harra* ethanolic extract was evaluated for its antioxidant potential using different in vitro tests such as 2,2-diphenyl-1-picrylhydrazyl, 2,2′-azino-bis(3-ethylbenzothiazoline-6-sulfonic acid), Ferric Reducing Ability Power, and *β*-carotene bleaching tests. The inhibitory activity of carbohydrate-hydrolyzing enzymes (α-amylase and α-glucosidase) and pancreatic lipase was also assessed. In the 2,2′-azino-bis(3-ethylbenzothiazoline-6-sulfonic acid assay, an IC_50_ value comparable to the positive control ascorbic acid (2.87 vs. 1.70 μg/mL, respectively) was obtained. The wild-wall rocket salad extract showed a significant α-amylase inhibitory effect. Obtained results indicate that Sicilian wild-wall rocket contains phytochemicals that can prevent hyperglycemia, hyperlipidemia, and obesity.

## 1. Introduction

According to data reported in 2016 by the Food and Agriculture Organization of the United Nations (FAO), more than 100 million Europeans have consumed wild foods [1]. The consumption of wild food is a global concept that mixes phenomena such as hunting, fishing, and gathering mushrooms and edible plants. Specifically in past decades, on the European continent, the consumption of wild herbs was very widespread due to the lifestyle and diet that were led [2]. Agri-food activities, such as livestock breeding, pastoralism, and cultivation of tree species, enormously influenced the botanical knowledge of the population that was in close contact with nature, making it possible to distinguish which species were edible and marketable. These include, for example, the Brassicaceae, one of the most important families within the circuit of edible plants and which today includes between 11 and 20 edible species [3]. This family includes several genera, such as *Brassica* (e.g., broccoli, cauliflower, mustard, etc.), *Raphanus* (e.g., radish and horseradish), *Wasabia* (wasabi) [4], and *Diplotaxis* (e.g., *tenuifolia* and *erucoides*). Obviously, not all these genera offer edible plants, but edible ones have been domesticated and are now widely used in cooking.

Plants of this last genus are characterized by having siliqua-shaped fruits (i.e., three times longer than wide), yellow flowers, and simple or absent hairs. The basal leaves are serrated or deeply divided, while the cauline leaves are not amplexicaul. Fruit is a pendulous siliqua with an elliptical section, equipped with a short beak without seeds of 1–2 mm when ripe, carried by a peduncle interrupted by a scale, with seeds arranged in two series under each valve. The genus, native to Mediterranean Europe and Macaronesia, is represented in Sicily by *Diplotaxis harra* subsp. *crassifolia* (Raf) Maire, *D. tenuifolia* (L.) DC, *D. erucoides* (L.) DC., *D. viminea* (L.) DC., and *D. muralis* (L.) DC [5]. 

Among these, *D. harra* subsp. *crassifolia* [Homotypic syn.s *Sinapis crassifolia* Raf.; *D. crassifolia* (Raf.) DC.; *Pendulina crassifolia* (Raf.) Willk.; *P. lagascana* (DC.) Amo; *P. intricata* Willk.; *P. webbiana* Willk.] is a suffruticose plant (Ch suffr) with stems that are generally woody at the base. The leaves are fleshy, glabrous, or bristly, with the lower ones more or less pinnate, while the upper ones are oblanceolate, linear, toothed, or more or less entire. Yellow petals, 6–10 mm, siliques of 2–3 × 12–60 mm, pendulum, carried by peduncles of 6–14 mm, interrupted by a short nodule, and extended by the 2 mm gynophore [6]. This plant is typical of the chalky, hilly, and rocky environments of Central and Southern Sicily; it is also present in Spain and NW Africa and is limited to Tunisia, Algeria, and Morocco [5].

Among the various species of the *Diplotaxis* genus, several are used in the culinary field. For example, *D. muralis* (L.) DC has a similar use to *D. tenuifolia* (L.) DC, and its potential as a green component for the preparation of soups is highlighted in Italy [7]. In turn, *D. erucoides* (L.) DC is used as a raw element for the preparation of salads in Malta [8], Sicily (Italy), and Spain, and as a cooked or boiled ingredient in several provinces of Sicily [9] and Tuscany. Flowers of *D. catholica* (L.) DC are reported in Spain as a culinary embellishment.

*D. harra* (Forsk.) Boiss. is an important fodder plant of the semi-arid regions of North Africa [10], but it finds enormous use as a medicinal plant in Tunisia and Algeria [11], and in Sicily, where it is present almost everywhere, it is also consumed cooked [subsp. *crassifolia* (Raf.) Maire] [12]. *D. acris* (Forsk.) Boiss. and *D. simplex* (L.) DC are plants used as fodder for grazing, but their use as a raw element in salads is confirmed in North Africa [13] and in Middle Eastern countries such as Iraq and Jordan [13].

The phytochemical investigation of this genus, and in particular of *D. harra* (commonly known as ‘wall rocket’), an annual species with thick and woody roots common in the sandy and stony valleys of the desert of Egypt and Tunisia [14,15], has indicated the clear presence of high levels of acids such as arachidonic and palmitic, nonadecane, stigmasterol, and *β*-sitosterol. The more volatile components have shown excellent biocidal activity against Gram-positive and Gram-negative bacteria and against several fungal strains. The same acids listed above, tested in mixtures, showed strong activity against selected bacteria compared to investigated yeasts and fungi [16]. The antibacterial activity was also confirmed by another study [17]. The dichloromethane extract of *D. harra* flowers has, in fact, shown excellent activity against bacteria such as *Staphylococcus aureus* and *Listeria monocytogenes*. From the preliminary (TLC), qualitative (RP-HPLC-DAD-ESI-MS^n^ and GC–MS), and separative analyses, the presence of a characteristic metabolite of this genus emerged: sulforaphane. This compound, belonging to the class of isothiocyanates, is known for its antitumor, antioxidant, and anti-inflammatory properties and was indicated for the first time as being responsible for the antibacterial properties of *D. harra* [17].

The interest in *D. harra* is, in fact, due to its use as an ethnomedicinal plant for the treatment of various pathologies such as diabetes, hypercholesterolemia, cancer, and anemia [18,19]. The high anti-inflammatory and antitumor activities shown by the extracts of the aerial parts of this plant can certainly be related to the notable content of polyphenols [20,21]. The excellent biological potential of this plant was also demonstrated in Muhammad et al. work [22]; in fact, the ethanolic extract of the apical parts was screened in vitro by evaluating the cytotoxicity activity against three different cell lines: HCT116, HepG2, and MCF-7, obtaining satisfactory IC_50_ values (4.65, 12.60, and 17.90 μg/mL, respectively). This polar extract was subjected to chromatographic analysis, and from the fractionation, five flavonoids were obtained, such as quercetin, quercetin 3-O-*β*-glucoside, isorhamnetin 7-O-*β*-glucoside, apigenin 3-O-*β*-rhamnoside, and kaempferol 3-O-*β*-glucoside, according to mass data and to comparison with literature data. The individual isolated metabolites tried in vitro and with the MTT test showed worse cytotoxic activity than that of the extract, confirming, in this case, the synergistic importance between different molecules for the improvement of antitumor activity [22]. Finally, the chloroform and the defatted aqueous methanol extracts showed positive antioxidant, cytotoxic, and antiviral effects. The extracts expressed the greatest antiproliferative activity against tumor lines such as P388 and MKN-28 with IC_50_ values of 0.40 and 0.07 mg/mL and against cancer cell line P388 with IC_50_ values of 0.24 and 0.25 mg/mL, respectively. The chloroform extract was found to be the most promising. In fact, it inhibited P388 eukaryotic DNA topoisomerase II with an IC_50_ of 0.24 mg/mL [21].

The prevalence of metabolic syndrome (MetS) is increasing significantly throughout the world. Patients affected by MetS are almost always obese. The adipose tissue promotes oxidative stress, which is involved in some typical manifestations of the pathology, namely insulin resistance and consequent type 2 diabetes (TDM2) and hyperlipidemia [23]. Healthy eating habits and the use of appropriate nutraceuticals with antioxidant, hypoglycemic, and hypolipidemic action could prevent or delay MetS [24].

Given the promising results obtained from studies of *D. harra*, the main aim of this work was to evaluate the phytochemical content of the subspecies of *D. harra* subsp. *crassifolia* (Raf.). Maire was never investigated to examine its biological potential as a fresh vegetable and nutraceutical product. For these reasons, only the aerial, ripe, and edible parts were taken into consideration in this work. An attempt was made to relate our results to the results of the previously mentioned taxonomic investigations. Finally, some biological aspects are discussed, such as the antioxidant activity, carbohydrate-hydrolyzing enzymes, and lipase inhibitory effects.

## 2. Results and Discussion

### 2.1. Diplotaxis harra subsp. Crassifolia Chemical Profile

**Figure 1:**
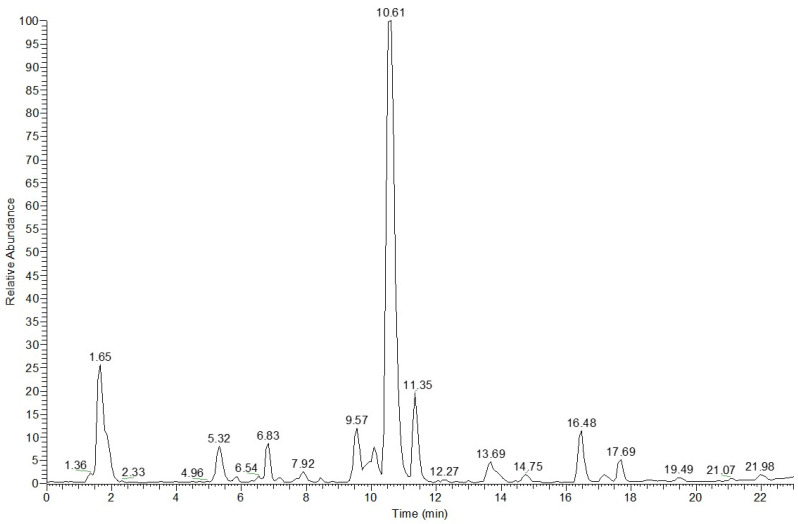
LC–ESI/HRMS profile of *D. harra* subsp. *crassifolia* ethanolic extract.

The results of the LC–ESI/HRMS^n^ analysis, comprising the assignment of molecular formula, the evaluation of the chromatographic elution order, and the study of the tandem mass spectra for each ion peak, indicated the occurrence in *D. harra* subsp. *crassifolia* ethanolic extract of several sulfated secondary metabolites, including glucosinolates and thirteen sulfooxy derivatives, along with flavonoids, hydroxycinnamic acid derivatives, and oxylipins (Figure 1, Table 1).

In agreement with a previous report on glucosinolates in *Diplotaxis* spp. [25], the hydroxybenzylglucosinolate sinalbin (**2**) resulted in the glucosinolate characterizing the analyzed species. In particular, the HRMSMS spectrum corroborated this assignment by providing the product ion at *m*/*z* 344.0806 (C_14_H_18_O_7_NS), which originated from the typical neutral loss of SO_3_ [(M-H)-80]^−^ from the molecular anion. Additionally, the typical product ions at *m*/*z* 274.9895 (C_6_H_11_O_8_S_2_) and *m*/*z* 259.0124 (C_6_H_11_O_9_S) were observed, originating from rearrangements and breakdowns involving the common structural skeleton of glucosinolates. The diagnostic product ion at *m*/*z* 261.9846 (C_8_H_8_O_5_NS_2_) promptly allowed the identification of the aglycon variable side chain as a hydroxybenzyl (Table 1).

Moreover, the occurrence of this glucosinolate was also proved by the detection of some metabolites likely generated from the metabolism of sinalbin, which, as it is well known, is hydrolyzed by enzymes (called myrosinases) into unstable aglycones that ultimately yield either isothiocyanates, nitriles, or other kinds of products [26]. Subsequently, nitrilases, bi-functional nitrile degrading enzymes, are involved in the simultaneous catalysis of two different reactions on a single substrate: the hydrolysis of nitriles to corresponding carboxylic acids and ammonia and the addition of water, forming stable amide end products, so acting as nitrile hydratases [26]. These considerations could support the finding of hydroxyphenylacetonitrile, hydroxyphenylacetamide, and hydroxyphenylacetic acid compounds in the form of sulfo-derivatives (**8**, **20**, and **24**, respectively) (Table 1). Only the sulfo-derivative of hydroxyphenylacetic acid has been previously described in Brassica oleracea [27]. Furthermore, in *D. harra* subsp. *crassifolia* extract, the (sulfo)hydroxybenzoic acid (**19**), a metabolite already described in the aforementioned report on *B. oleracea*, could be detected [27]. The sulfated compound **6** could be assigned as a (sulfoglycosyl)hydroxybenzoic acid or a (sulfo)hydroxybenzoate-glycoside (Table 1), depending on the glycosylation site, considering that both metabolites, in the form with no sulfo group, have been previously described in Brassicaceae [28,29,30,31,32]. Analogously, compounds **4**, **9**, and **11** could be tentatively assigned as both sulfated and glycosylated derivatives of hydroxyphenylacetic acid, never described until now in the Brassicaceae family, neither in this form nor in the glycosylated one (Table 1), whereas a diglycosilated form of the hydroxybenzoic acid has been reported in *B. napus* [29]. Another metabolite, compound **18**, was tentatively identified as (sulfo)sinalbin, being characterized by the occurrence of an additional sulfur atom in its molecular formula and yielding a tandem mass spectrum showing as the base peak the product ion at *m*/*z* 424.0372 (C_14_H_18_O_10_NS_2_), corresponding to sinalbin and obtained by neutral loss of an SO_3_ molecule from the [M-H]^−^ ion (Table 1). This metabolite is described for the first time in a member of the Brassicaceae family, but other sulfoglucosinolates have been reported in the family, e.g., N-sulfoglucobrassicin [33]. Compounds **12**, **17**, **23**, **25**, and **27** could be likely defined as sulfo-derivatives of C13 nor-isoprenoids, a class of metabolites previously reported as no-sulfated compounds, e.g., in *B. fruticulosa* [34], and as sulfated compounds in plants such as cress [35].

The LC–ESI/HRMS^n^ analysis allowed us to assign compounds **10**, **16**, **21**, **22**, **3**, **13**, **7**, and **14** as glycosides of isorhamnetin, quercetin, and kaempferol, respectively (Table 1). This assignment is in agreement with both mass spectrometric data from the literature and previous reports on *Diplotaxis* spp., highlighting compounds **3** and **7** as never described in *D. harra* [36,37,38,39,40,41,42]. 

On the basis of their chromatographic elution order and of the occurrence of the product ion at *m*/*z* 163.0401 (C_9_H_7_O_3_) and *m*/*z* 173.0454 (C_7_H_9_O_5_), respectively, as a base peak in their tandem mass spectra [43], compounds **5** and **15** could be identified as 3-*p*-coumaroylquinic acid and 4-*p*-coumaroylquinic acid, respectively (Table 1), two metabolites already described in genera of the Brassicaeae family other than *Diplotaxis* [44,45]. Finally, compounds **26** and **28** could be identified as two oxilipins previously reported in *D. erucoides* (Table 1) [37]. 

### 2.2. Antioxidant Activity

The antioxidant activities of *D. harra* subsp. *crassifolia* EtOH extract were studied, and data are reported in Table 2. Except for the FRAP test, the sample showed concentration-dependent activity. As unexpected, different behavior of EtOH extract was observed in radical scavenging assays (DPPH and ABTS). 

In fact, a 1.7-time lower radical scavenging activity was observed against ABTS^+·^ when the extract was tested in comparison to the positive control ascorbic acid (IC_50_ value of 2.87 vs. 1.70 μg/mL, respectively). The DPPH test is characterized by a lower sensitivity than the ABTS assay when D. harra extract was tested with an IC_50_ value of 46.99 μg/mL. It is possible to suppose that wild-wall rocket salad contains phytochemicals that are able to release hydrogen ions into ABTS free radicals. A low Ferric Reducing Ability Power was found with an FRAP value of 12.55 μM Fe(III)/g. Good protection from lipid peroxidation was observed at both incubation times, with IC_50_ values of 21.62 and 35.19 μg/mL after 30 and 60 min of incubation, respectively. The radical scavenging potential of *D. harra* subsp. *crassifolia* was better than those found for *D. erucoides* subsp. *erucoides*, collected in the same geographical area, with IC_50_ values of 135.13 and 97.87 μg/mL for DPPH and ABTS, respectively [37]. In the same order of potency against DPPH radical were Tunisian *D. virgata* and *D. erucoides* extracts with IC_50_ values from 20.01 to 40.05 μg/mL and from 24.01 to 27.02 μg/mL, respectively. The same trend was not observed against ABTS radical scavengers; a lower activity was observed in comparison with our sample [46].

### 2.3. Carbohydrate Hydrolyzing and Lipase Enzymes Inhibitory Activities

The search for natural products able to counteract hyperglycemia and hyperlipidemia is a very important research topic for the growing number of patients suffering from these pathologies. *D. harra* subsp. *crassifolia* extract exhibited inhibited carbohydrate hydrolyzing and lipase enzymes in a concentration-dependent manner. Data are reported in Table 3. 

Generally, *α*-amylase resulted in more sensitivity to the action of wild-wall rocket extract with an IC_50_ value of 139.52 μg/mL, which is only 2.66 lower than the positive control acarbose (IC_50_ value of 52.44 μg/mL). An IC_50_ value of 242.32 μg/mL was found against *α*-glucosidase. A limited pancreatic lipase inhibitory activity was observed in comparison to the positive control orlistat (IC_50_ values of 799.66 vs. 37.42 μg/mL, respectively). The Sicilian wild-wall rocket resulted in less activity in comparison to *D. erucoides* subsp. *erucoides* [37]. A similar consideration could be made against pancreatic lipase (IC_50_ value of 61.27 μg/mL). IC_50_ values of 346 and 46 μg/mL were found for *D. simplex* flower extract against *α*-amylase and *α*-glucosidase, respectively [47]. The activity of *Diplotaxis* in hyperglycaemic and hyperlipidaemic patients was confirmed by an in vivo study where administration of *D. simplex* (200 mg/kg 1 body mass) for 30 days restored the normal glycemia and serum lipid profile in diabetic rats [47]. 

## 3. Materials and Methods

### 3.1. Chemicals and Reagents

Methanol, ethanol, FeCl_3_, HCl, CH_3_COONa, CH3COOH, CHCl_3_, NaNO_2_, NaOH, Na_2_CO_3_, AlCl_3_, FeSO_4_, NaCl, NaH_2_PO_4_, Na_2_HPO_4_, K_2_S_2_O_8_, HClO_4_, and HCl were obtained from VWR International s.r.l. (Milano, Italy). Water, acetonitrile, and formic acid used for liquid chromatography–mass spectrometry (LC–MS) were of the Merck brand and were purchased from Deltek (Naples, Italy). 2,2-Diphenyl-1-picrylhydrazyl (DPPH), 2,2′-azino-bis(3-ethylbenzothiazoline-6-sulfonic acid) (ABTS), tripyridyltriazine (TPTZ), *β*-Carotene, ascorbic acid, butylated hydroxytoluene (BHT), propyl gallate, quercetin, chlorogenic acid, linolenic acid, Tween 20, potato starch, sodium potassium tartrate, 3,5-dinitro salicylic acid, *α*-amylase (EC 3.2.1.1), *α*-glucosidase (EC 3.2.1.20), maltose, *o*-dianisine, PGO Enzyme Preparation, porcine pancreatic lipase (EC 3.1.1.3), *p*-nitrophenyl carryate, TRIZMA, DMSO, acarbose, orlistast, Folin–Ciocalteu reagent, were purchased from Sigma-Aldrich S.p.a. (Milano, Italy). All the reagents used in the study were of analytical grade.

### 3.2. Plant Material and Extraction Procedure

The apical tops and young leaves of *D. harra* subsp. *crassifolia* (Raf.) Maire, not in the flowering state, were collected between Marianopoli and Santa Caterina Villarmosa, in the province of Caltanissetta (Sicily, Italy) (37°59′28″ N, 14°01′62″ E, 605 m above sea level), at the beginning of April 2020. An herbarium specimen (PAL109805) was identified and deposited in the herbarium of the University of Palermo by Prof. Vincenzo Ilardi. The aerial parts were dried and pulverized by using an electric blender. Approximately 150 g of dried plant was immersed in absolute ethanol (≥99.8%) and extracted sequentially three times with the same amount of solvent. The extract was then filtered, and the filtrates were concentrated using a rotary evaporator to give a solid residue equal to a yield of 5.38%. 

### 3.3. Total Phenols and Flavonoids Content

The total phenol content (TPC) was monitored using the spectrophotometric method proposed by Folin–Ciocalteu, as previously reported [48]. TPC was expressed as mg of gallic acid equivalents (GAE)/g of fresh sample.

The total flavonoid content (TFC) was determined using spectrophotometric quantification of the flavonoid–aluminum complex [48]. TFC was expressed as mg quercetin equivalents (QE)/g of fresh sample. 

### 3.4. LC–ESI/HRMS^n^ Analysis

The ethanolic extract of *D. harra* subsp. *crassifolia* was analyzed by a system of liquid chromatography coupled to electrospray ionization and high-resolution mass spectrometry (LC–ESI/HRMS, ThermoScientific, San Jose, CA, USA) composed of a quaternary Accela 600 pump and an Accela autosampler coupled to an LTQ Orbitrap XL mass spectrometer (ThermoScientific, San Jose, CA, USA), operating in negative electrospray ionization mode. The separation was carried out on a Symmetry RP-18 (2.1 × 150 mm, 5 μm; Waters, Milford, MA, USA) column at a flow rate of 0.2 mL/min. The mobile phase consisted of a combination of A (0.1% formic acid in water, *v*/*v*) and B (0.1% formic acid in acetonitrile, *v*/*v*). A linear gradient from 10 to 50% B in 20 min and to 100% B in 3 min was used. The autosampler was set to inject 2 μL of extract (0.5 mg/mL). The following experimental conditions for the ESI source were adopted: sheath gas flow at 15 (arbitrary units); auxiliary gas flow at 5 (arbitrary units); capillary temperature at 280 °C; source voltage at 3.5 kV; capillary voltage at −48 V; and tube lens at −176.47 V. The mass range was from *m*/*z* 200 to 800, with a resolution of 30,000. The first and second most intense ions occurring in the high-resolution mass spectrum (HRMS) were selected in data-dependent scan experiments to perform collision-induced dissociation (CID) by applying a minimum signal threshold of 250, an isolation width of 2.0, and a normalized collision energy of 30%. Xcalibur software version 2.1 was used for instrument control, data acquisition, and data analysis. For each sample, three replicates were performed.

### 3.5. Antioxidant Activity of D. harra subsp. crassifolia Extracts

The antioxidant activity of *D. harra* subsp. *crassifolia* extracts was investigated by using different in vitro tests. 2,2-Azino-bis(3-ethylbenzothiazoline-6-sulfonic acid) (ABTS) and 1,1-diphenyl-2-picrylhydrazyl (DPPH) radical scavenging activity were used to investigate the radical scavenging potential, whereas the ability of the sample to reduce ferric ions was studied by using the Ferric Reducing Ability Power (FRAP) test. The *β*-carotene bleaching test was performed to investigate the ability of the extract to protect from lipid peroxidation. All test protocols were previously reported [48]. Briefly, in the DPPH test, a solution of DPPH was mixed with extract at different concentrations (10–1000 μg/mL). After that, the mixture was incubated at room temperature for 30 min. The absorbance was read at 517 nm. In the ABTS test, a solution of ABTS radical cation was prepared and, after 12 h, was diluted with ethanol to reach an absorbance of 0.70 at 734 nm. The stabilized ABTS^+^ solution was added to extracts at different concentrations (1–400 μg/mL), and the mixture was left to react at room temperature for 6 min. After that, the absorbance was read at 734 nm. Ascorbic acid was employed as a positive control in both the ABTS and DPPH assays. In the FRAP test, a mixture of tripyridyltriazine (TPTZ) solution, FeCl_3_, and acetate buffer was added to the sample at a concentration of 2.5 mg/mL. After 30 min of incubation at room temperature, the absorbance was measured at 595 nm. Butylated hydroxytoluene (BHT) was used as a positive control [48]. The protection from lipid peroxidation was studied by using the *β*-carotene bleaching test. Briefly, a solution of *β*-carotene, linoleic acid, and Tween 20 was prepared and added to the samples at different concentrations (5–100 μg/mL). The mixture was left to react for 30 and 60 min at 45 °C, and the absorbance was read at 470 nm. Propyl gallate was used as a positive control [48].

### 3.6. Carbohydrates-Hydrolyzing Enzymes and Pancreatic Lipase Inhibitory Activity

The *α*-amylase inhibitory activity was assessed as previously described [25]. Briefly, a starch solution was added to the extract at different concentrations (25–1000 μg/mL) and left to react with the enzyme (EC 3.2.1.1) for 5 min at room temperature. After that, the absorbance was read at 540 nm. In the *α*-glucosidase inhibitory assay, a maltose solution was mixed with *α*-glucosidase (EC 3.2.1.20), peroxidase/glucose oxidase system-color reagent, and *o*-dianisidine solution. Then, the extract (25–1000 μg/mL) was added to the prepared solution and left to react for half an hour at 37 °C. The absorbance was read at 500 nm. Acarbose was used as the positive control in both tests.

The pancreatic lipase inhibition activity was assessed as previously described [49]. The extract at different concentrations (2.5–40 mg/mL) was mixed with the enzyme (EC 232-619-9), 4-nitrophenyl octanoate, and left to react at 37 °C for 30 min. After that, the absorbance was measured at 405 nm. Orlistat was used as a positive control. 

### 3.7. Statistical Analyses

Each experiment was performed in triplicate and repeated three times. Data are expressed as means ± standard deviation (S.D.). The concentration–response curve and the inhibitory concentration of 50% (IC_50_) were calculated using Prism GraphPad Prism version 4.0 for Windows, GraphPad Software (San Diego, CA, USA). One-way analysis of variance (ANOVA) followed by a multicomparison Dunnett’s test (*** *p* < 0.001) by using Prism GraphPad Prism version 4.0 for Windows, GraphPad Software (San Diego, CA, USA).

## 4. Conclusions

The present study assessed the phytochemical profile, antioxidant activity, α-amylase, α-glucosidase, and lipase inhibitory effect of wild-wall rocket salad, *D. harra* subsp. *crassifolia*, growing in Sicily (Italy). The LC–ESI/HRMS^n^ analysis of the ethanolic extract highlighted the occurrence of flavonoids, hydroxycinnamic acid derivatives, and oxylipins, but above all of several sulfated secondary metabolites, among which sulfo-derivatives of C13 nor-isoprenoids and hydroxyphenyl and hydroxybenzoic acids, and the glucosinolate (sulfo)sinalbin (**18**), metabolites that in most cases have never been reported before in the Brassicaeae family or in the *Diplotaxis* genus. Promising ABTS radical scavenging potential and α-amylase inhibitory effects were recorded. The antioxidant activity demonstrated, mainly due to the high presence of hydroxylated compounds, would also explain the beneficial use of this plant, or other ssp., in traditional medicine for the treatment of inflammatory processes, irritations, and intestinal disorders. Moreover, the in-depth phytochemical profile could help to support the identification of the large number of local varieties to counter the globalization of agricultural production that would lead to a restricted range of varieties grown with a concrete threat to biodiversity. Further study could be conducted to identify single compounds responsible for the bioactivity found.

## Figures and Tables

**Table 1:** Metabolites were identified in *D. harra* subsp. *crassifolia* ethanolic extract.

N.	Compound	R_t_ (min)	Molecular Formula	[M-H]^−^	RDB	Delta ppm	HRMSMS
**1**	Disaccharide	1.65	C_12_H_22_O_11_	341.1085	2.5	1.91	179.0561 (C_6_H_11_O_6_)
**2**	Sinalbin	5.32	C_14_H_19_O_10_NS_2_	424.0373	6.5	1.55	344.0806 (C_14_H_18_O_7_NS); 274.9895 (C_6_H_11_O_8_S_2_); 261.9846 (C_8_H_8_O_5_NS_2_); 259.0124 (C_6_H_11_O_9_S)
**3**	Quercetin 3,7-*O*-diglycoside	5.86	C_27_H_30_O_17_	625.1404	13.5	0.82	505.0979 (C_23_H_21_O_13_); 463.0873 (C_21_H_19_O_12_); 462.0796 (C_21_H_18_O_12_); 343.0460 (C_17_H_11_O_8_); 301.0348 (C_15_H_9_O_7_)
**4**	(Sulfoglycosyl)hydroxyphenylacetic acid/(sulfo)hydroxyphenylacetate-glycoside	6.32	C_14_H_18_O_11_S	393.0492	6.5	1.56	273.0065 (C_10_H_9_O_7_S); 230.9965 (C_8_H_7_O_6_S)
**5**	3-*p*-Coumaroylquinic acid	6.39	C_16_H_18_O_8_	337.0922	8.5	1.26	163.0401 (C_9_H_7_O_3_)
**6**	(Sulfoglycosyl)hydroxybenzoic acid/(sulfo)hydroxybenzoate-glycoside	6.54	C_13_H_16_O_11_S	379.0329	6.5	−0.23	299.0769 (C_13_H_15_O_8_); 259.0124 (C_6_H_11_O_9_S); 216.9810 (C_7_H_5_O_6_S)
**7**	Kaempferol 3,7-*O*-diglycoside	6.62	C_27_H_30_O_16_	609.1452	13.5	0.31	489.1027 (C_23_H_21_O_12_); 447.0922 (C_21_H_19_O_11_); 285.0395 (C_15_H_9_O_6_)
**8**	(Sulfo)hydroxyphenylacetamide	6.69	C_8_H_9_O_5_NS	230.0122	5.5	1.96	150.0559 (C_8_H_8_O_2_N)
**9**	(Sulfoglycosyl)hydroxyphenylacetic acid/(Sulfo)hydroxyphenylacetate-glycoside	6.75	C_14_H_18_O_11_S	393.0493	6.5	1.88	273.0068 (C_10_H_9_O_7_S); 230.9965 (C_8_H_7_O_6_S)
**10**	Isorhamnetin 3,4′-*O*-diglycoside	6.83	C_28_H_32_O_17_	639.1553	13.5	−0.48	519.1138 (C_24_H_23_O_13_); 477.1031 (C_22_H_21_O_12_); 357.0608 (C_18_H_13_O_8_); 315.0507 (C_16_H_11_O_7_)
**11**	(Sulfodiglycosyl)hydroxyphenylacetic acid/(Sulfo)hydroxyphenylacetate-diglycoside	7.15	C_20_H_28_O_16_S	555.1013	7.5	−0.20	393.0490 (C_14_H_17_O_11_S); 375.0383 (C_14_H_15_O_10_S)
**12**	(Sulfo)tetrahydroxy-megastigmene	7.92	C_13_H_24_O_7_S	323.1167	2.5	2.57	96.9598 (HSO_4_)
**13**	Quercetin 3,4′-*O*-diglycoside	8.20	C_27_H_30_O_17_	625.1406	13.5	1.11	463.0864 (C_21_H_19_O_12_); 301.0346 (C_15_H_9_O_7_)
**14**	Kaempferol-3,4′-*O*-diglycoside	8.22	C_27_H_30_O_16_	609.1456	13.5	0.92	447.0918 (C_21_H_19_O_11_); 285.0398 (C_15_H_9_O_6_)
**15**	4-*p*-Coumaroylquinic acid	8.37	C_16_H_18_O_8_	337.0921	8.5	0.91	173.0454 (C_7_H_9_O_5_)
**16**	Isorhamnetin 3,7-*O*-diglycoside	8.44	C_28_H_32_O_17_	639.1553	13.5	−0.38	519.1128 (C_24_H_23_O_13_); 477.1033 (C_22_H_21_O_12_); 476.0955 (C_22_H_20_O_12_); 357.0609 (C_18_H_13_O_8_); 315.0507 (C_16_H_11_O_7_); 313.0356 (C_16_H_9_O_7_)
**17**	(Sulfo)tetrahydroxy-megastigmene	9.57	C_13_H_24_O_7_S	323.1167	2.5	2.47	96.9598 (HSO_4_)
**18**	(Sulfo)sinalbin	9.94	C_14_H_19_O_13_NS_3_	503.9936	6.5	0.22	424.03719 (C_14_H_18_O_10_NS_2_); 261.9842 (C_8_H_8_O_5_NS_2_)
**19**	(Sulfo)hydroxybenzoic acid	10.09	C_7_H_6_O_6_S	216.9809	5.5	3.7	172.9914 (C_6_H_5_O_4_S); 137.0246 (C_7_H_5_O_3_); 96.9598 (HO_4_S)
**20**	(Sulfo)hydroxyphenylacetic acid	10.61	C_8_H_8_O_6_S	230.9964	5.5	2.80	187.0065 (C_7_H_7_O_4_S); 151.0400 (C_8_H_7_O_3_); 107.05064 (C_7_H_7_O)
**21**	Isorhamnetin 4′-*O*-diglycoside	10.98	C_28_H_32_O_17_	639.1562	13.5	1.05	315.0504 (C_16_H_11_O_7_); 300.0268 (C_15_H_8_O_7_); 271.0235 (C_14_H_7_O_6_)
**22**	Isorhamnetin 3-*O*-glycoside	11.35	C_22_H_22_O_12_	477.1028	12.5	0.25	449.1079 (C_21_H_21_O_11_); 387.0716 (C_19_H_15_O9); 357.0613 (C_18_H_13_O_8_); 315.0506 (C_16_H_11_O_7_); 314.0429 (C_16_H_10_O_7_); 299.0192 (C_15_H_7_O_7_); 285.0399 (C_15_H_9_O_6_); 271.0242 (C_14_H_7_O_6_); 257.0451 (C_14_H_9_O_5_); 243.0299 (C_13_H_7_O_5_); 151.0038 (C_7_H_3_O_4_)
**23**	(Sulfo)trihydroxy-megastigmadiene/Epoxy-(sulfo)dihydroxy-megastigmene	13.69	C_13_H_22_O_6_S	305.1055	3.5	0.64	96.9598 (HSO_4_)
**24**	(Sulfo)hydroxyphenylacetonitrile	14.75	C_8_H_7_O_4_NS	212.0017	6.5	2.29	132.0456 (C_8_H_6_ON)
**25**	(Sulfo)trihydroxy-megastigmadiene/Epoxy-(sulfo)dihydroxy-megastigmene	15.12	C_13_H_22_O_6_S	305.1057	3.5	1.33	96.9599 (HSO_4_)
**26**	9,12,13-Trihydroxyoctadeca-10,15-dienoic acid	16.48	C_18_H_32_O_5_	327.2172	3.5	1.89	309.2069 (C_18_H_29_O_4_); 291.1962 (C_18_H_27_O_3_); 239.1284 (C_13_H_19_O_4_); 229.1443 (C_12_H_21_O_4_); 221.1181 (C_13_H_17_O_3_); 211.1339 (C_12_H_19_O_3_); 171.1026 (C_9_H_15_O_3_)
**27**	(Sulfo)dihydroxy-megastigmadienone	17.16	C_13_H_20_O_6_S	303.0902	4.5	1.63	96.9599 (HSO_4_)
**28**	9,12,13-Trihydroxyoctadec-10-enoic acid	17.69	C_18_H_34_O_5_	329.2327	2.5	1.46	311.2222 (C_18_H_31_O_4_); 293.2116 (C_18_H_29_O_3_); 229.1442 (C_12_H_21_O_4_); 211.1337 (C_12_H_19_O_3_); 171.1028 (C_9_H_15_O_3_)

**Table 2:** In vitro antioxidant activity of *D. harra* subsp. *crassifolia* EtOH extract.

Sample	DPPH Test IC_50_ (μg/mL)	ABTS Test IC_50_ (μg/mL)	FRAP ^#^ Test μM Fe(II)/g	*β*-Carotene Bleaching Test IC_50_ (μg/mL)	
				30 min	60 min
*D. harra*	46.99 ± 5.96 ***	2.87 ± 3.81 ^ns^	12.55 ± 1.38 ***	21.62 ± 3.73 ***	35.19 ± 1.49 ***
Positive controls					
Ascorbic acid	5.0 ± 0.8	1.7 ± 0.06	-	-	-
BHT	-	-	63.27 ± 4.35	-	-
Propyl gallate	-	-		1.04 ± 0.04	0.09 ± 0.00

Data are given as media ± S.D. (*n* = 3); ^#^ at 2.5 mg/mL; ascorbic acid. BHT and propyl gallate were used as positive controls in antioxidant tests. Differences within and between groups were evaluated by one-way ANOVA followed by a multicomparison Dunnett’s test (*** *p* < 0.0001); ^ns^: not significant.

**Table 3:** Carbohydrates hydrolyzing enzymes and pancreatic lipase inhibitory activities (IC_50_, μg/mL) of *D. harra* subsp. *crassifolia* extract.

Sample	*α*-Amylase	*α*-Glucosidase	Pancreatic Lipase
*D. harra*	139.52 ± 4.09 ***	242.32 ± 10.12 ***	799.66 ± 17.22 ***
Positive control			
Acarbose	52.44 ± 3.91	35.33 ± 3.76	-
Orlistat	-	-	37.42 ± 1.05

Data are expressed as mean ± S.D. (*n* = 3). Differences within and between groups were evaluated by a one-way ANOVA followed by a multicomparison Dunnett’s test (*** *p* < 0.001).

## Data Availability

Data will be made available from the corresponding author upon reasonable request.

## Abbreviations

ABTS, 2,2′-azinobis(3-ethylbenzothiazoline-6-sulfonic) acid; BHT, hydroxytoluene; CAE, chlorogenic acid equivalents; DPPH, 2,2-diphenyl-1-picrylhydrazyl; FFA, fatty free acids; FRAP, Ferric Reducing Ability Power; GLs, glucosinolates; IC_50_, inhibitory concentration 50%; LC–ESI/HRMS, liquid chromatography coupled to electrospray ionization and high-resolution mass spectrometry; NPC, 4-nitrophenyl octanoate; PGO, peroxidase/glucose oxidase; QE, quercetin equivalents; S.D., standard deviation; TD2, type 2 diabetes; TFC, total flavonoid content; TPC, total phenol content; TPTZ, tripyridyltriazine.
